# Correction to: Dynamic switch of immunity and antitumor effects of metformin in rat spontaneous esophageal carcinogenesis

**DOI:** 10.1007/s00262-021-03039-7

**Published:** 2021-09-07

**Authors:** Ryohei Takei, Tomoharu Miyashita, Satoshi Takada, Hidehiro Tajima, Itasu Ninomiya, Hiroyuki Takamura, Sachio Fushida, Ai Harashima, Seiichi Munesue, Shintaro Yagi, Noriyuki Inaki, Tetsuo Ohta, Yasuhiko Yamamoto

**Affiliations:** 1 Department of Gastroenterologic Surgery, Kanazawa University Graduate School of Medical Sciences, Kanazawa, 920-8640 Japan; 2 Department of Surgical Oncology, Kanazawa Medical University Hospital, 13-1 Takaramachi, Kanazawa, 920-8640 Japan; 3 Department of Biochemistry and Molecular Vascular Biology, Kanazawa University Graduate School of Medical Sciences, Kanazawa, 920-8640 Japan

## Correction to: Cancer Immunology, Immunotherapy 10.1007/s00262-021-03027-x

The original version of this article unfortunately contained a mistake in Fig. 6. The corrected Fig. 6 is given in the next page.

**Fig. 6:**
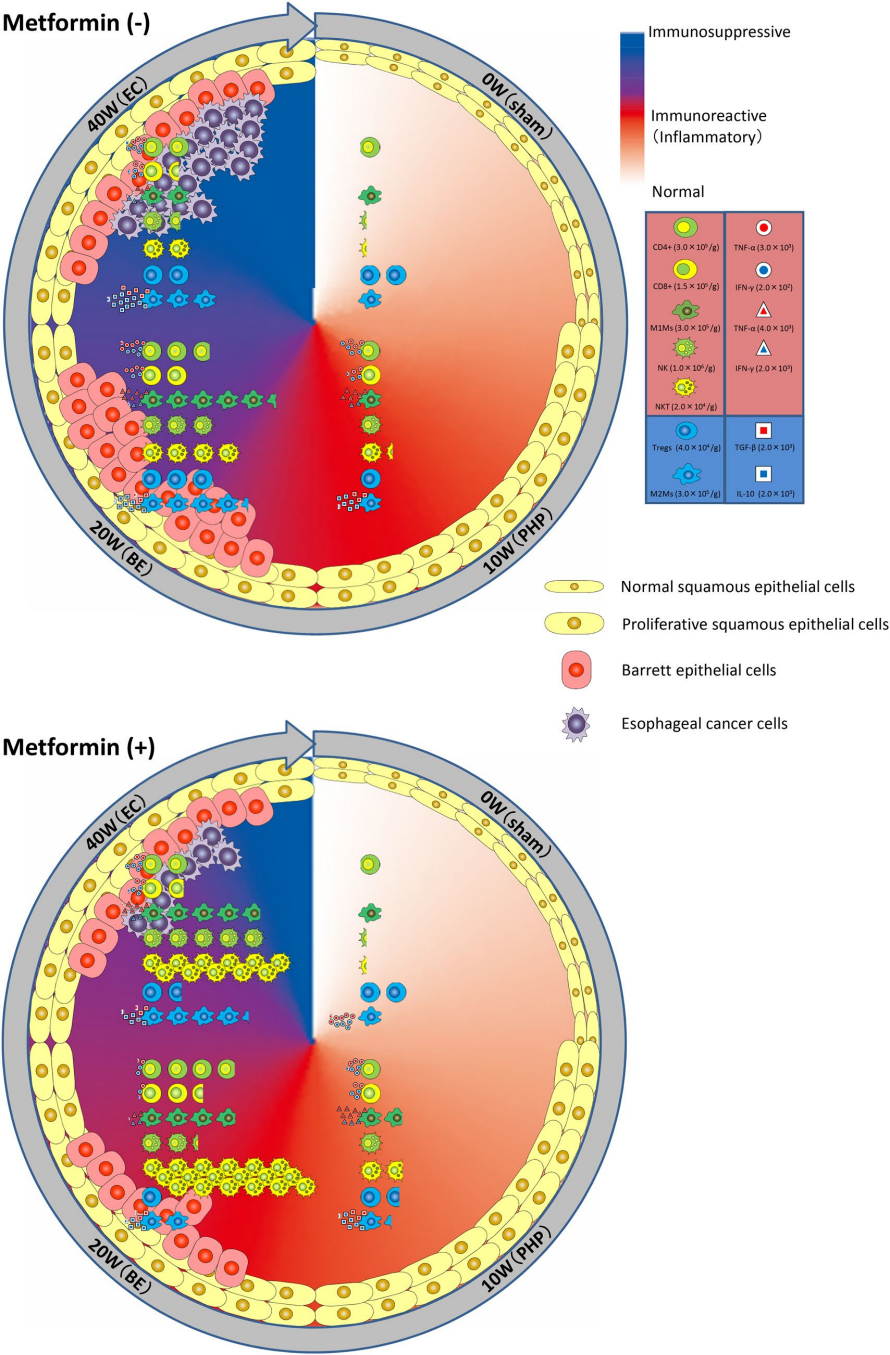
Illustrated here are dynamic changes of immune cell populations and characteristics during the carcinogenic transition from gastroesophageal reflux disease (GERD) to Barrett’s esophagus (BE) and finally to esophageal cancer (EC) in the esophagus of this rat model. Inflammatory reaction is initiated around 10 weeks after the surgery and proliferative hyperplasia (PHP) of the esophagus epithelial cells is observed. Metformin could impact the modulation of pro-inflammatory reactions in esophageal carcinogenesis and host antitumor immunity by improving the immunosuppressive tumor microenvironment and immune evasion

